# Supplementary material for: Stacking Model‐Based Classifiers for Dealing With Multiple Sets of Noisy Labels
Source: Biom J. 2025 Mar 12;67(2):e70042. doi: 10.1002/bimj.70042 (PMC11898607; doi:10.1002/bimj.70042)
Supplement: Supplementary file 1 — Supporting Information [file BIMJ-67-e70042-s001.zip › density-based-ensemble-model-main/competitors_real_data/netlab-master/netlab-master/help/demmet1.htm]

Netlab Reference Manual demmet1


# demmet1

## Purpose

Demonstrate Markov Chain Monte Carlo sampling on a Gaussian.

## Synopsis

```
demmet1
demmet1(plotwait)
```

## Description

The problem consists of generating data from a Gaussian
in two dimensions using a Markov Chain Monte Carlo algorithm. The points are
plotted one after another to show the path taken by the chain.

`demmet1(plotwait)` allows the user to set the time (in a whole number
of seconds) between the plotting of points. This is passed to `pause`

## See Also

`demhmc1`, `metrop`, `gmm`, `dempot`

---

**Pages:**
Index


---

Copyright (c) Ian T Nabney (1996-9)
